# Development of a novel screening questionnaire for brain fog

**DOI:** 10.1192/j.eurpsy.2024.773

**Published:** 2024-08-27

**Authors:** Y. Jheng, W.-C. Hsieh, Y.-H. Chou

**Affiliations:** ^1^Center for Quality Management; ^2^Department of Psychiatry, Taipei Veterans General Hospital; ^3^Department of Psychiatry, School of Medicine, National Yang-Ming Chiao-Tung University, Taipei, Taiwan

## Abstract

**Introduction:**

Amidst the widespread proliferation of the COVID-19 virus, brain fog has become one of the most critical issues of public health. Brain fog may lead to sub-health conditions, such as forgetfulness, difficulty thinking, and other related symptoms. Although they are not immediately life-threatening, these sub-health conditions could gradually erode the quality of life. Currently, there is no relevant screening tool for brain fog.

**Objectives:**

The aim of this study was to develop a reliable screening tool.

**Methods:**

A web-based brain fog screening questionnaire was developed in the study. It was based on previous studies, which summarized five parts of the most common clinical symptoms after COVID-19: forgetfulness, difficulty thinking, difficulty concentrating, feeling confused, and difficulty finding words or phrases to speak. Unfortunately, these items were used only in a way of yes or no answers in previous studies. Each of these items was expanded to five anchors to evaluate their severity in the study. Cronbach’s alpha coefficient was used to assess internal consistency. K-means clustering was used as a second method to validate the cutoff points. Furthermore, the receiver operating characteristic (ROC) curve was applied to validate the appropriateness of the cutoff point.

**Results:**

There were 534 participants who completely finished the questionnaire. It includes 183 males and 351 females, and all of them aged between 19 and 81 years. The Cronbach’s Alpha value was 0.821. The cutoff point was at a total score of 6 in terms of K-means. Based on the result, the ROC curve revealed that an area under the curve (AUC) was 0.816 with a confidence interval of 0.784 to 0.849.

**Conclusions:**

The study demonstrated the feasibility and reliability of the web-based screening test for brain fog.

**Disclosure of Interest:**

None Declared

